# Transcriptome and pan-cancer system analysis identify PM2.5-induced stanniocalcin 2 as a potential prognostic and immunological biomarker for cancers

**DOI:** 10.3389/fgene.2022.1077615

**Published:** 2023-01-06

**Authors:** Dong Zhu, Jiliu Liu, Junyi Wang, Lei Zhang, Manling Jiang, Yao Liu, Ying Xiong, Xiang He, Guoping Li

**Affiliations:** ^1^ State Key Laboratory of Quality Research in Chinese Medicine, Macau University of Science and Technology, Taipa, Macau SAR, China; ^2^ Laboratory of Allergy and Precision Medicine, Chengdu Institute of Respiratory Health, The Third People’s Hospital of Chengdu, Affiliated Hospital of Southwest Jiaotong University, Chengdu, China; ^3^ Department of Pulmonary and Critical Care Medicine, Chengdu Third People’s Hospital Branch of National Clinical Research Center for Respiratory Disease, Affiliated Hospital of ChongQing Medical University, Chengdu, China; ^4^ Department of Pulmonary and Critical Care Medicine, Sichuan Friendship Hospital, Chengdu, China

**Keywords:** PM2.5, prognosis, biomarker, cancer, stanniocalcin 2 (STC2)

## Abstract

Epidemiological studies have shown that air pollution and particulate matter (PM) are closely related to the occurrence of cancer. However, the potential prognostic and immunological biomarkers for air pollution related cancers are lacking. In this study, we proved PM2.5 exposure was correlated with lung cancer through transcriptome analysis. Importantly, we identified STC2 as a key gene regulated by PM2.5, whose expression in epithelial cells was significantly increased after PM2.5 treatment and validated by using RT-qPCR and immunofluorescence. Kaplan-Meier OS curves suggested that high STC2 expression positively correlated with a poor prognosis in lung cancer. Furthermore, we discovered that STC2 was associated with multiple cancers and pathways in cancer. Next, Pan-Cancer Expression Landscape of STC2 showed that STC2 exhibited inconsistent expression across 26 types of human cancer, lower in KIRP in cancer versus adjacent normal tissues, and significantly higher in another cancers. Cox regression results suggested that STC2 expression was positively or negatively associated with prognosis in different cancers. Moreover, STC2 expression was associated with clinical phenotypes including age, gender, stage and grade. Mutation features of STC2 were also analyzed, in which the highest alteration frequency of STC2 was presented in KIRC with amplification. Meanwhile, the effects of copy number variation (CNV) on STC2 expression were investigated across various tumor types, suggesting that STC2 expression was significantly correlated with CNV in tumors. Additionally, STC2 was closely related to tumor heterogeneity, tumor stemness and tumor immune microenvironment like immune cell infiltration. In the meantime, we analyzed methylation modifications and immunological correlation of STC2. The results demonstrated that STC2 expression positively correlated with most RNA methylation genes and immunomodulators across tumors. Taken together, the findings revealed that PM2.5-induced STC2 might be a potential prognostic and immunological biomarker for cancers related to air pollution.

## Introduction

Exposure to high concentration of air pollutants causes public health problems. It has been reported that ambient air pollution is linked to serious adverse health outcomes like incidence and mortality of cardiovascular, pulmonary diseases, and even cancer (Gourd, 2022; He et al., 2022; Joseph et al., 2022). The International Agency for Research on Cancer (IARC) classified outdoor air pollution and the particulate matter (PM) as carcinogens, based on sufficient evidence on the carcinogenicity to humans and experimental animals (WHO, 2013). Particulate matter of 2.5 μm or less in diameter (PM2.5) is one of the most complex pollutants in the atmospheric environment. People’s concern about the effect of environmental pollution, especially PM2.5 on cancer has increased year by year. Epidemiological studies have also shown that PM2.5 is closely related to the occurrence of cancer (Pun et al., 2017; Yu et al., 2021; Bo et al., 2022). However, the potential prognostic and immunological biomarkers for air pollution related cancers are lacking.

As the leading cause of mortality in the world, cancer brings a great economic burden to the society, and it seriously affects people’s quality of life and life cycle (Nathan et al., 2022; Shih et al., 2022). The treatments of tumors mainly include surgery, chemotherapy, radiotherapy, targeted therapy and immunotherapy (Jiang et al., 2022; Mauz-Körholz et al., 2022; Perone and Anaya, 2022). Although these treatment methods have achieved some success to a certain extent, the prognosis and survival rate of patients are still unsatisfactory due to the complexity of tumors and the characteristics of gene mutations. Therefore, it is urgent to actively search for other new therapeutic targets and tumor biomarkers to improve the effect of tumor diagnosis and treatment, especially related to PM2.5 exposure. With the continuous improvement of public databases like The Cancer Genome Atlas (TCGA), it is possible to discover new therapeutic targets and biomarkers by performing pan-cancer analysis of genes and evaluation of the correlations between specific genes and clinical prognosis or related signaling pathways.

Stanniocalcin 2 (STC2) encodes a secreted, homodimeric glycoprotein that is expressed in different organs and tissues. This gene is involved in regulating a wide variety of pathophysiological processes due to the autocrine or paracrine functions (Kobberø et al., 2022). Studies have showed that the expression of STC2 is broadly increased in human tumors including renal cell carcinoma, breast cancer, and colorectal cancer (Esseghir et al., 2007; Meyer et al., 2009; Miyazaki et al., 2014). Meanwhile, it has been reported that STC2 is induced by PM2.5 exposure, which is correlated with epithelial-mesenchymal transition (EMT) markers (Zhang et al., 2021). In this study, we identified STC2 as a key gene regulated by PM2.5 based on transcriptome analysis, whose expression correlated to prognosis, immune microenvironment, tumor heterogeneity and stemness, immune checkpoint genes, epigenetic modifications in multiple cancer types.

## Materials and methods

### Reagents

Anti-STC2 antibody (Abcam, ab255610), Goat Anti-rabbit IgG H&L (Alexa Fluor 555) (Abcam, ab150078). 2×Taq SYBR Green qPCR Mix (Vazyme, SQ101), HiScript IIQ RT SuperMix for qPCR (Vazyme, R223–01), FastPure Cell/Tissue Total RNA Isolation Kit (Vazyme, RC101–01). PM2.5 sample was prepared as previously described (Xiang et al., 2020). Briefly, the PM2.5 samples were collected using air sampler. The filters were preheated at 550°C, then balanced and stabilized. Then the sampled filters were cut into small pieces, immersed followed by sonication. The extracted PM2.5 components were lyophilizated, stored at -80°C until use.

### Cell culture

Human normal lung epithelial cell (BEAS-2B) was purchased from Shanghai Jikai gene Chemistry Technology Co., Ltd. The Bease-2B cells were cultured with Dulbecco’s Modified Eagle’s Medium (DMEM) culture medium containing 10% fetal bovine serum (FBS) (Gibco, United States) and 1% penicillin-streptomycin mixed solution at 37°C in with 5% CO_2_ condition. After BEAS-2B cells reached 60% confluence in plates, 62 μg/cm2 of PM2.5 was added to cell cultures for 24 h.

### Quantitative PCR analysis of RNA expression

The total RNA in BEAS-2B cells and tissue was extracted by FastPure Cell/Tissue Total RNA Isolation Kit (Vazyme, RC101–01). The concentration and purity of extracted RNA were (260/280 ratio) was determined using ScanDrop (Analytik Jena AG, GER), and then converted into cDNA with a Reverse Transcription Kit on Kit (Vazyme, R223–01) following the manufacturer’s protocol. Quantitative PCR was performed with SYBR solution purchased from Vazyme (SQ101) according to the manufacturer’s protocols.

### RNA-seq

Transcriptome librariy was prepared as previously described (Liu et al., 2022). Briefly, mRNA was isolated from 5 μg of total RNA by oligo (dT) beads followed by fragmentation and cDNA was synthesized. The synthesized cDNA was subjected to end-repair, phosphorylation and “A” base addition according to Illumina’s library construction protocol. The size of library was selected followed by PCR amplified for 15 PCR cycles. Paired-end sequencing was performed with the Illumina HiSeq xten by BGI Company. Then clean reads were separately aligned to reference genome with orientation mode using TopHat.

### Confocal microscopy and image analysis

BEAS-2B cells were seeded on cover glass, stabilized for 24 h, and then treated with PM2.5 for 24 h. Cells were fixed with cold 10% paraformaldehyde in PBS for 30 min at 4°C, and then incubated with STC2 antibodies (1:100) at 4°C overnight. Alexa Fluor 555-conjugated goat anti-Rabbit IgG H&L (1:2,000) were used to bind primary Abs for 1 h at root temperature. After washed vigorously three times with PBS, Nuclei were stained with 4′-6-diamidino-2-phenyl-indole dihydrochloride (DAPI) for 5 min. The cover glasses were washed with PBS three times and placed on glass slides before confocal microscopy. Images were taken on a OLMPUS confocal microscopy (CSU-W1, Japan).

### Gene set enrichment analysis

Gene Expression Series (GSE155616, GSE158954, and GSE182199) were downloaded from the National Center of Biotechnology Information (NCBI) Gene Expression Omnibus Database (GEO, http://www.ncbi.nlm.nih.gov/geo/). The gmt files of genesets were downloaded from MSigDB (http://www.gsea-msigdb.org). Differentially expressed genes were analyzed by using the limma R package. Gene set enrichment analysis was performed with GESA 3.0.

### Correlation of STC2 expression with prognosis, and clinical phenotypes

Data of clinical phenotypes were extracted for each sample downloaded from TCGA. Overall survival (OS), disease-specific survival (DSS), disease-free interval (DFI), and progression-free interval (PFI), were selected to study the relationship between STC2 expression and patient prognosis. The Kaplan-Meier method and log-rank test were used for survival analyses (*p* < 0.05) of each cancer type. Survival curves and cox analysis were performed using the R packages “survival,” “survminer,” and “forestplot.”

### Tumor immune microenvironment

The correlation between STC2 expression and the abundance of immune infiltration in pan-cancer were analyzed using TIMER, CIBERSORT, EPIC database and MCP-counter R package. To determine the effects of SCT2 on the immune microenvironment in various tumor types, the stromal and immune scores of tumors were evaluated using the ESTIMATE algorithm.

### Genetic alteration analysis of STC2

STC2 genetic alteration were analyzed by using cBioPortal (https://www.cbiopo.rtal.org/). The alteration frequency of amplification, mutation and deep deletion across all TCGA tumors were observed.

### Correlation of STC2 expression with tumor heterogeneity and stemness

The data of TMB, MSI, purity, ploidy and tumor stemness index was obtained from previous publications (Bonneville et al., 2017; Malta et al., 2018; Thorsson et al., 2018). The correlation between STC2 expression and TMB, MSI, purity, ploidy and tumor stemness index was evaluated using the Pearson correlation coefficient. Differences with a *p*-value <0.05 were considered to be statistically significant.

### Correlation between STC2 expression and RNA methylation modifications, immunomodulators, immune checkpoints genes

The relationship between STC2 expression and RNA methylation related genes, immunomodulators, immune checkpoints genes was evaluated by SangerBox tools (Shen et al., 2022).

## Results

### PM2.5 exposure is associated with lung cancer

**FIGURE 1:**
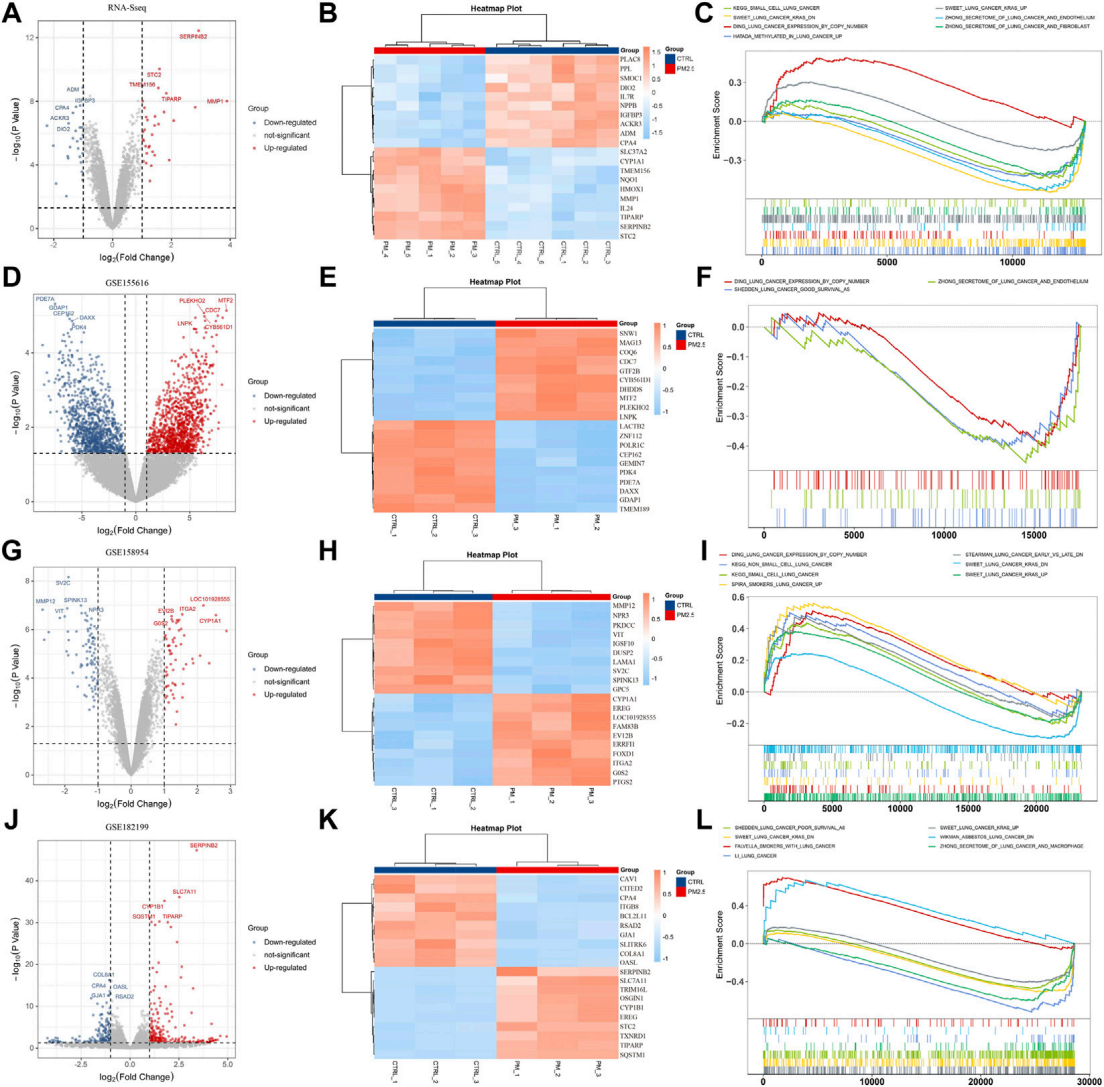
PM2.5 exposure is associated with lung cancer. **(A,D,G,J)** Volcano plots showing DEGs from our own RNA-seq data and public datasets (GSE155616, GSE158954, and GSE 128199); **(B,E,H,K)** Heatmaps showing top 10 DEGs. **(C,F,I,L)** GSEA analysis showing PM2.5 exposure was associated with lung cancer.

To identify the differentially expressed genes in response to PM2.5 exposure, human normal bronchial epithelial cells (BEAS-2B) were treated with PM2.5, and RNA-seq was performed subsequently. It showed that PM2.5 induced significant gene expression alterations including STC2 (Figures 1A,B). Furthermore, Gene Set Enrichment Analysis (GSEA) explored that PM2.5 exposure was related to KEGG_SMALL_CELL_LUNG_CANCER, SWEET_LUNG_CANCER_KARS, DING_LUNG_CANCER_EXPRESSION_BY_COPY_NUMBER, suggesting that PM2.5 exposure was associated with lung cancer (Figure 1C). Moreover, we collected three PM2.5-exposed datasets from public database. Consistent with the analysis based on our own RNA-seq data, GSEA analysis showed that PM2.5 exposure was involved in varieties of lung cancer gene sets (Figure 1D–L).

### STC2 is a key gene regulated by PM2.5 and associated with multiple cancers

**FIGURE 2:**
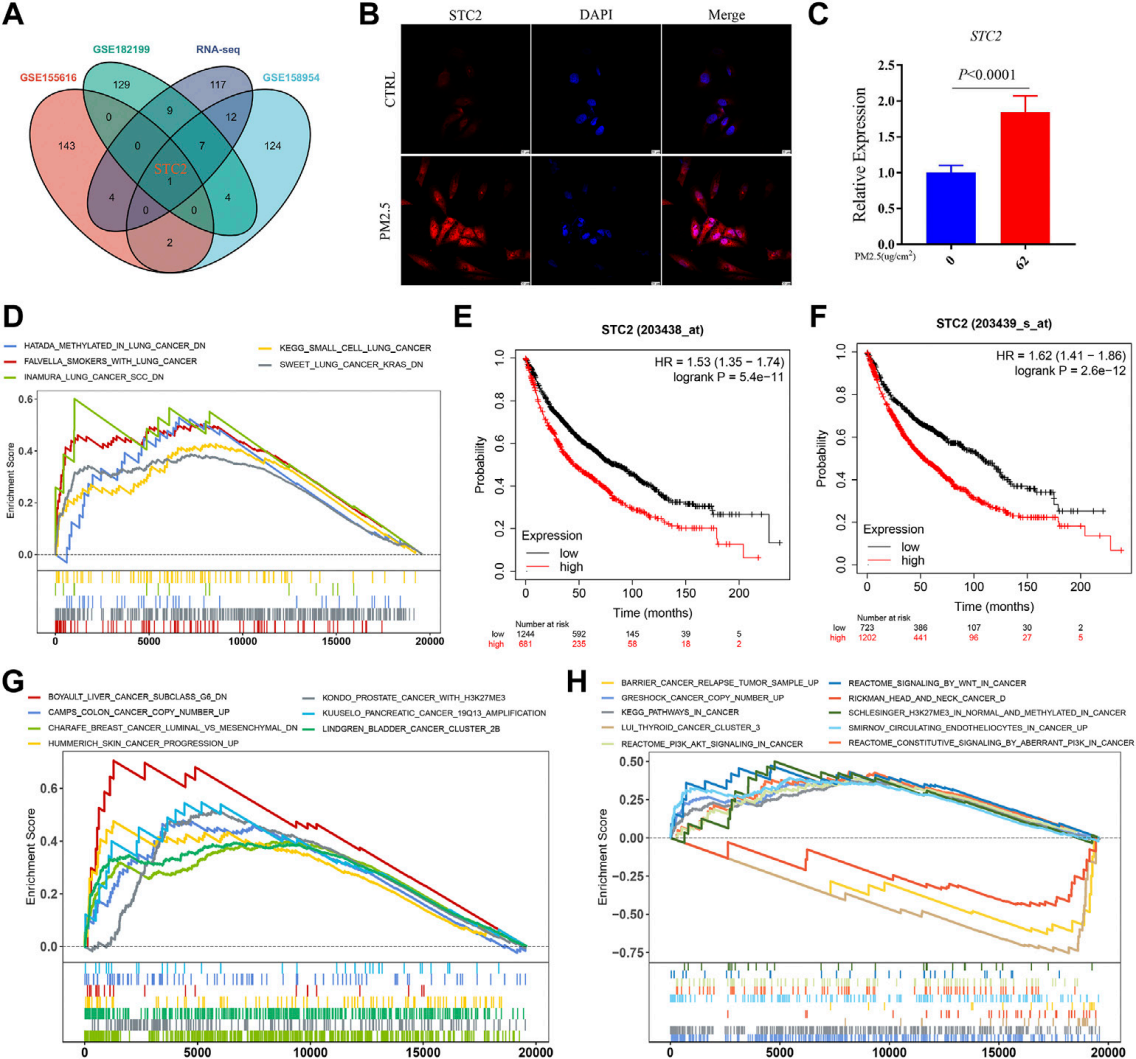
STC2 is a key gene regulated by PM2.5 and associated with multiple cancers. **(A)** Venn plot showing PM2.5 induced STC2 in different gene sets; **(B)** Immunofluorescence imaging showing the expression level of STC2 in different group; **(C)** qPCR showing the relative expression of STC2 in different group; **(D)** GSEA analysis showing high STC2 expression associated with lung cancer; **(E,F)** Kaplan-Meier OS curves showing high STC2 expression positively correlated with a poor prognosis in lung cancer; **(G,H)** STC2 expression was associated with a variety of cancers.

STC2, the key gene regulated by PM2.5 was screened out by crossing the top 150 DEGs in each datasets (Figure 2A). The expression of STC2 was validated by using immunofluorescence and qPCR (Figures 2B,C). The results showed that the protein and transcript levels of STC2 were up-regulated by PM2.5 exposure. Then we collected the lung data from TCGA database. Based on STC2 expression levels, the samples were divided into two groups (high- and low-expression groups). GSEA analysis displayed that high-expression group was positively associated with lung cancer gene sets (Figure 2D). Meanwhile, Kaplan-Meier OS curves suggested that high STC2 expression positively correlated with a poor prognosis in lung cancer (Figures 2E,F). Importantly, we found that STC2 was associated with multiple cancers including liver cancer, colon cancer, breast cancer, skin cancer and so on (Figures 2G,H).

### Pan-Cancer Expression Landscape of STC2

**FIGURE 3:**
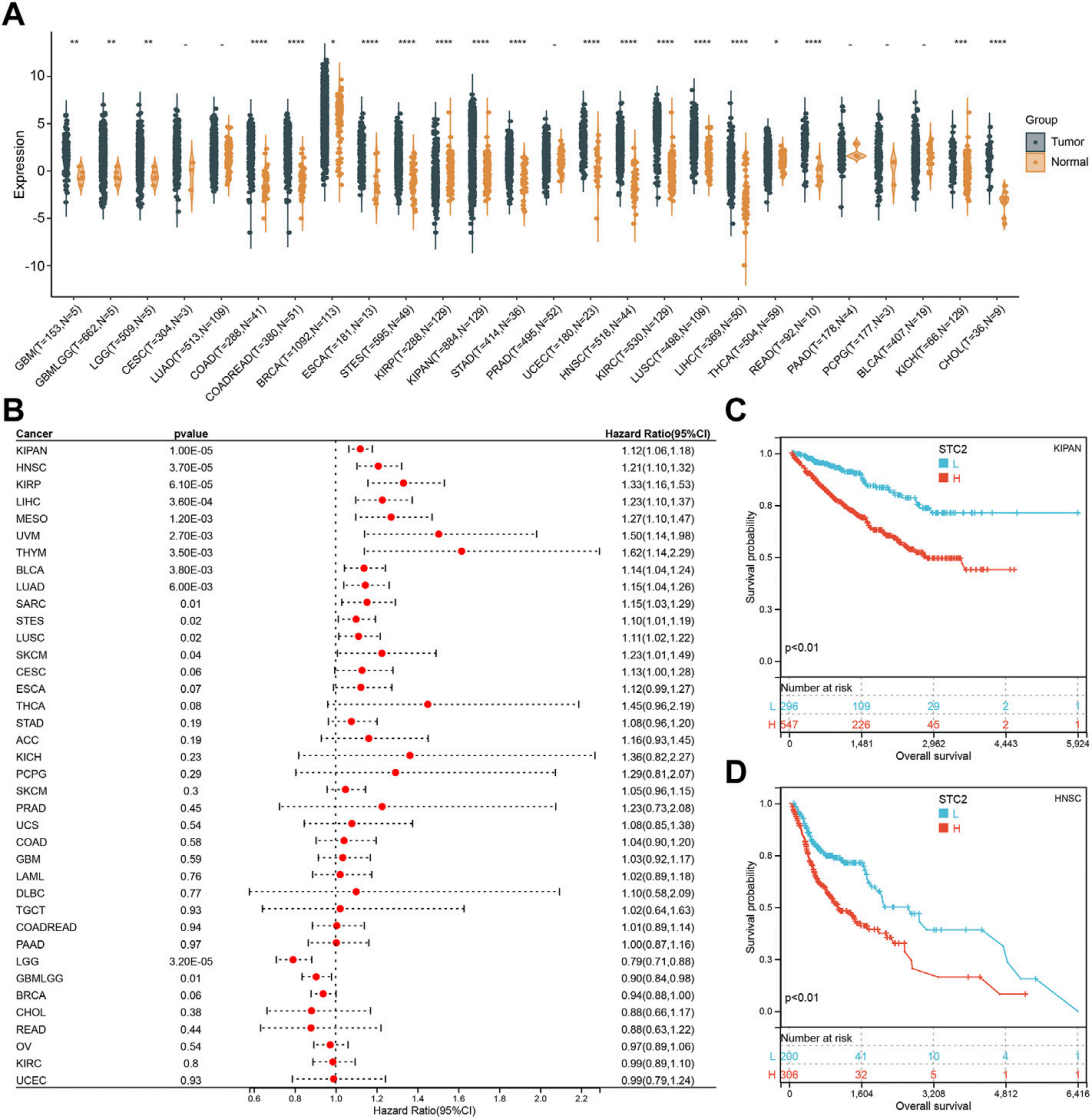
Pan-Cancer Expression Landscape of STC2. **(A)** STC2 expression across 26 types of human cancer; **(B)** Cox regression showing the correlation between STC2 expression and OS; **(C,D)** Two presentative Kaplan-Meier OS curves (KIPAN and HNSC). *: *p*-value < 0.05; **: *p*-value < 0.01, ***: *p*-value < 0.001, ****: *p*-value < 0.0001.

To explore the role of PM2.5-regulated STC2 in different cancers, we analyzed STC2 expression. According to the data from the TCGA database, STC2 exhibited inconsistent expression across 26 types of human cancer. Full name for the abbreviationof cancer was showed in Supplementary Table S1. The STC2 expression was lower in KIRP in cancer versus adjacent normal tissues, and significantly higher in another cancers except for CESC, LUAD, PRAD, PAAD, PCPG, and BLCA (*p* > 0.05) (Figure 3A). Meanwhile, we assessed the association between STC2 expression and the prognosis of patients. In OS analysis, Cox regression results suggested that high STC2 expression positively correlated with a poor prognosis for KIPAN, LIHC, HNSC, KIRP, MESO, THM, UVM, LUAD, BLCA, SARC, STES, ESCA, and SKCM, low STC2 expression negatively correlated with a poor prognosis for LGG and GBMLGG (Figure 3B). The STC2 expression also affected PFI on 14 types of cancer including KIRP, KIRPAN, BLCA, HNSC, THYM, CESC, ACC, KICH, MESO, UVM, LUAD, LGG, BRCA, GBMLGG, and THCA (Supplementary Figure S1A). For DFI, Cox regression analysis demonstrated that the increased STC2 expression was a risk factor for KIRP, ACC and CESC, and was protective factor for DLBC (Supplementary Figure S1B). Cox regression of DSS identified that low STC2 expression was a risk factor LGG, BRCA and GBMLGG, and was protective factor for KIRP, KIRPAN, HNSC, LIHC, CESC, UVM, ESCA, LUSC, THYM, BLCA, STES, and MESO (Supplementary Figure S1C). Two presentative Kaplan-Meier OS curves (KIPAN and HNSC) were showed in Figures 3C,D.

### Correlation between STC2 expression and clinical phenotypes

**FIGURE 4:**
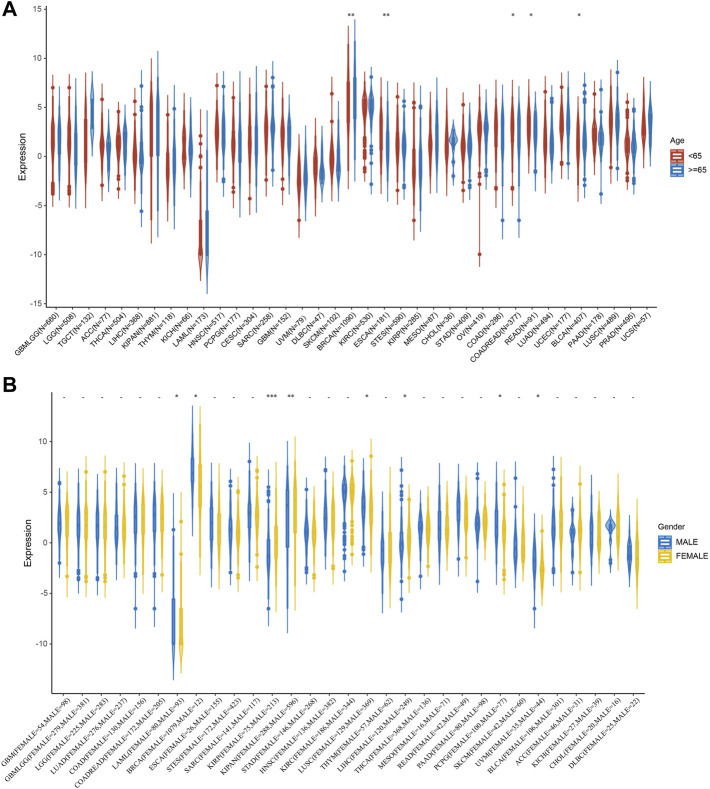
Correlation between STC2 Expression and Clinical phenotypes. **(A)** The relationship between STC2 expression and age; **(B)** The relationship between STC2 expression and gender. *: *p*-value < 0.05; **: *p*-value < 0.01, ***: *p*-value < 0.001.

**FIGURE 5:**
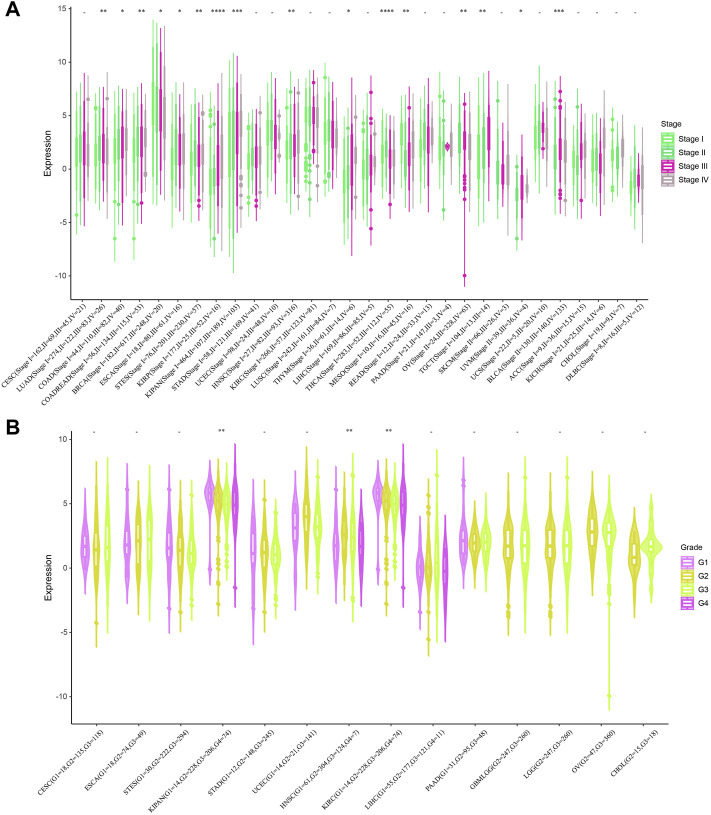
Correlation between STC2 Expression and Clinical phenotypes. **(A)** The relationship between STC2 expression and Stage; **(B)** The relationship between STC2 expression and Grade. *: *p*-value < 0.05; **: *p*-value < 0.01, ***: *p*-value < 0.001, ****: *p*-value < 0.0001.

**FIGURE 6:**
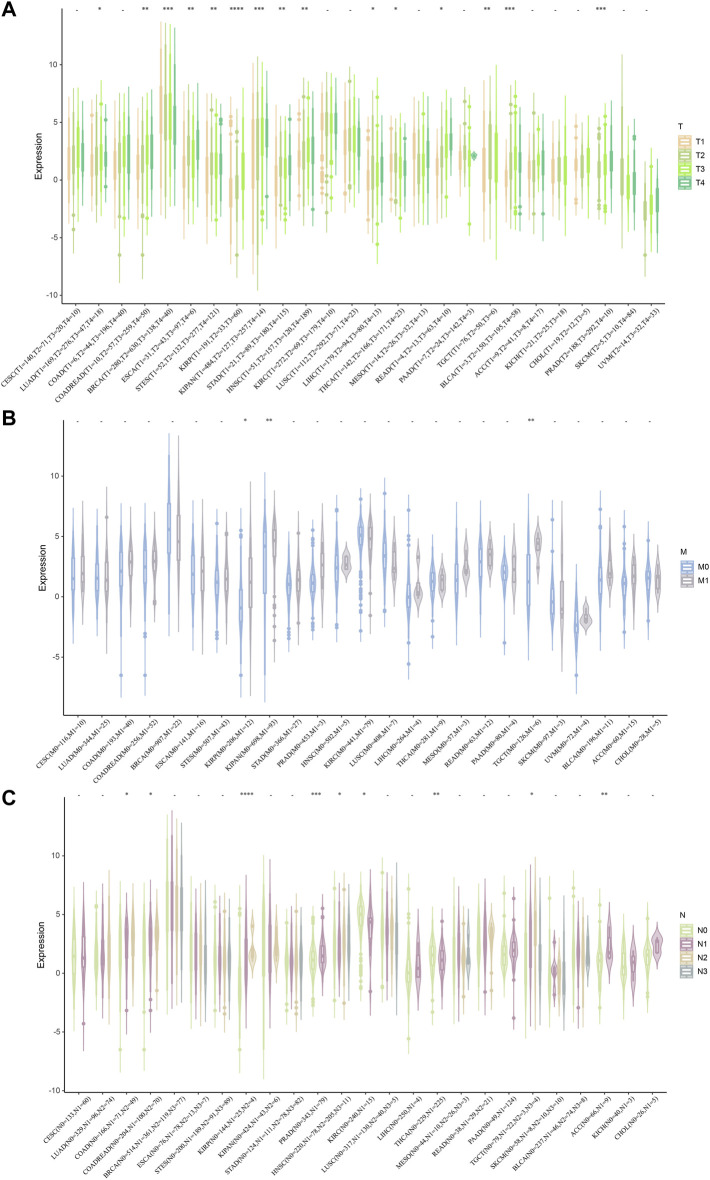
Correlation between STC2 Expression and Clinical phenotypes. **(A–C)** The relationship between STC2 expression and TMN staging. *: *p*-value < 0.05; **: *p*-value < 0.01, ***: *p*-value < 0.001, ****: *p*-value < 0.0001.

Next, we investigated the relationship between STC2 expression and clinical features in various cancers, including age, gender, stage, grade and TMN staging in patients with tumors. Firstly, we found that STC2 expression was significantly higher in patients older than 65 years in BRCA and BLCA than that in those younger than 65 years, but was lower in ESCA, COADREAD, and READ (Figure 4A). No significant correlations between age and STC2 expression were observed in patients with other cancers. Moreover, there was a clear gender difference in the expression of STC2 in LAML, BRCA, KIRP, KIPAN, LUSC, LIHC, PCPG, and UVM. In LAML, BRCA LUSC, PCPG, and UVM, STC2 were highly expressed in males than those in females, while it were less expressed in males than those in females with KIRP, KIPAN and LIHC cancers (Figure 4B). The relevance of tumor stage and grade were analyzed as well (Figures 5A,B). It showed that STC2 expression significantly correlated with tumor stage in majority of tumor types like KIRP and THCA, and was associated with tumor grade in KIPAN, HNSC, and KIRC. For TMN staging, the differential expression of STC2 was related to T stage in 15 types of cancer, including STES, KIRP, HNSC, and THCA (Figure 6A). Differences of STC2 expression were also observed in N stage and M stage across the cancers, nine types of cancer in N stage and only three types of cancer in M stage (Figures 6B,C).

### STC2 expression correlated with immune infiltration

**FIGURE 7:**
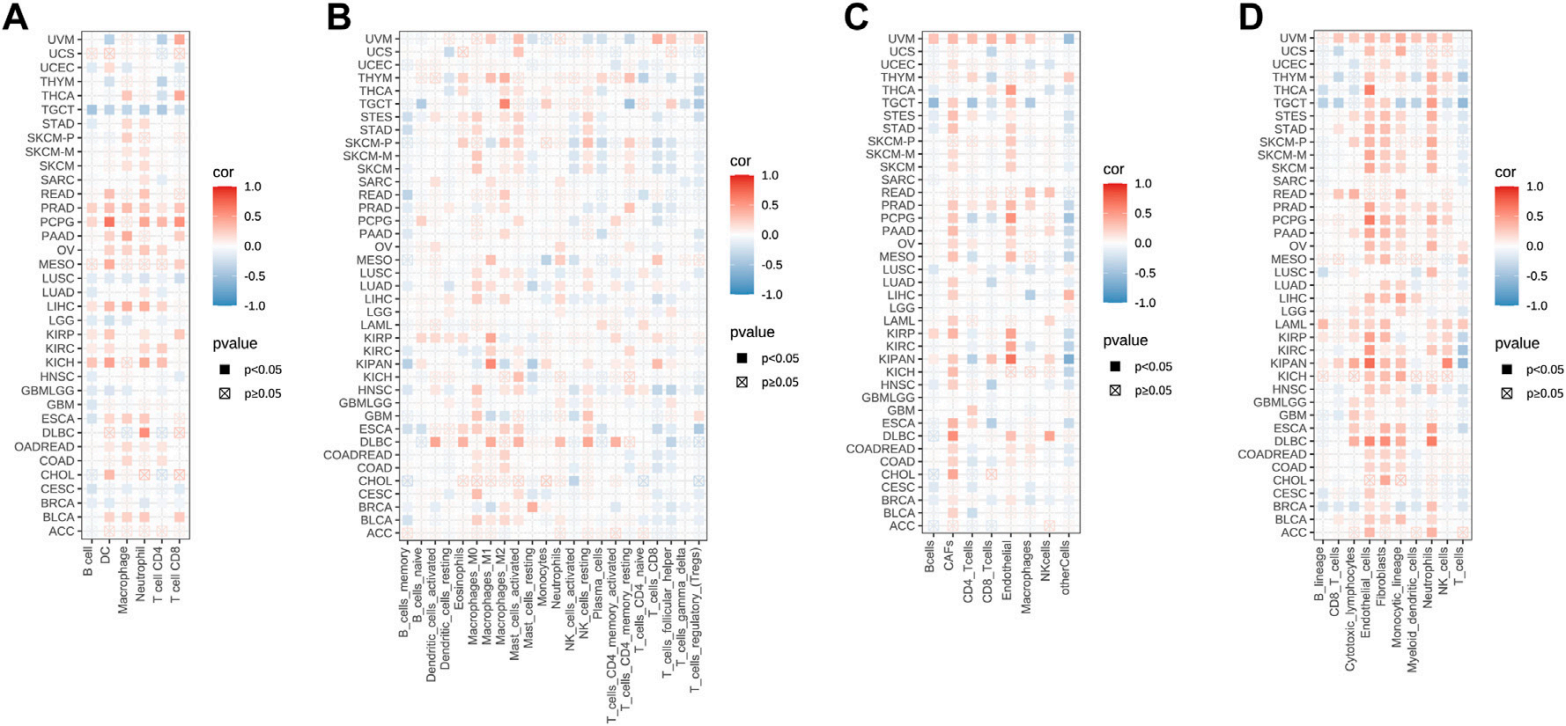
STC2 expression correlated with immune infiltration. **(A)** TIMER analysis showing the correlation between STC2 expression and immune cells infiltration; **(B)** CIBERSORT showing the correlation between STC2 expression and immune cells infiltration; **(C)** EPIC showing the correlation between STC2 expression and immune cells infiltration; **(D)** MCPcounter showing the correlation between STC2 expression and immune cells infiltration.

We next investigated the potential association between the infiltration level of immune cells and STC2 gene expression in diverse cancer types by using TIMER (Figure 7A). It showed that STC2 expression significantly correlated to the level of immune cells infiltration in most caner types. The results also displayed that STC2 expression was positively associated with all of the immune cells in PRAD, and negatively associated with all of the immune cells in TGCT. Meanwhile, by using CIBERSORT, we further examined the relationship between STC2 expression and the infiltration of immune cell subtypes (Figure 7B). Among 22 subtypes of immune cells, we observed a statistically positive correlation between STC2 expression and immune cells including M0, M1, and M2 macrophages, activated mast cells, and found the expression of SCT2 was negatively related to memory B cells in most cancer types. In CHOL, only activated NK cells was found negatively correlated with STC2 expression. Both methods, EPIC and MCPcounter, revealed that STC2 expression was positively associated with endothelial cells and fibroblasts across majority of tumors (Figures 7C,D).

**FIGURE 8:**
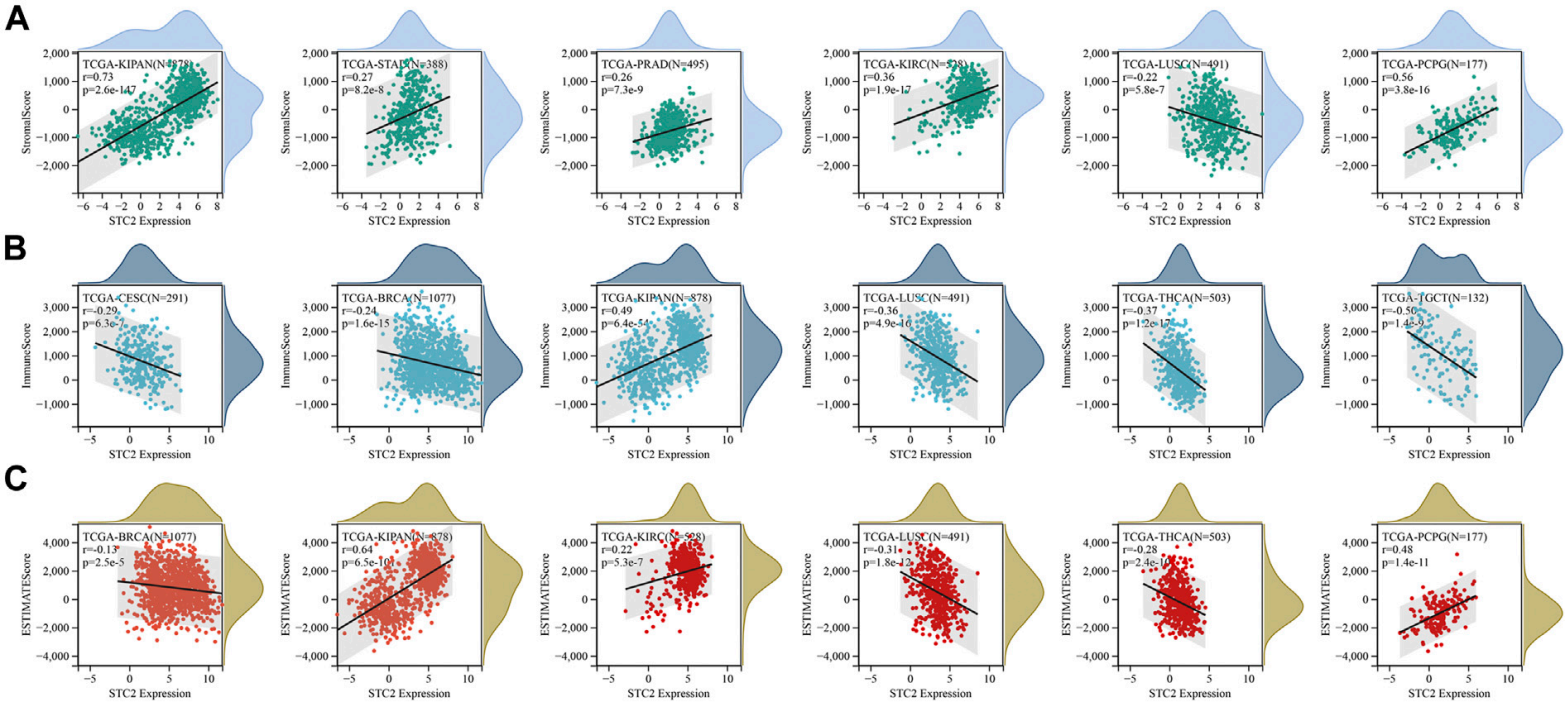
STC2 expression correlated with immune infiltration. **(A)** Correlation between STC2 expression and stromal score in six cancer types; **(B)** Correlation between STC2 expression and immune score in six cancer types; **(C)** Correlation between STC2 expression and estimate score in six cancer types.

Furthermore, the immune, stromal and estimate score was calculated by using ESTIMATE method (Figures 8A–C). Six cancer types with the lowest *p*-value in stromal score, immune score and estimate score were displayed, respectively. The results revealed that STC2 expression was positively associated with estimate score in KIPAN, KIRC, and PCPG, and negatively in BRCA, LUSC, and THCA.

### Mutation features of STC2

**FIGURE 9:**
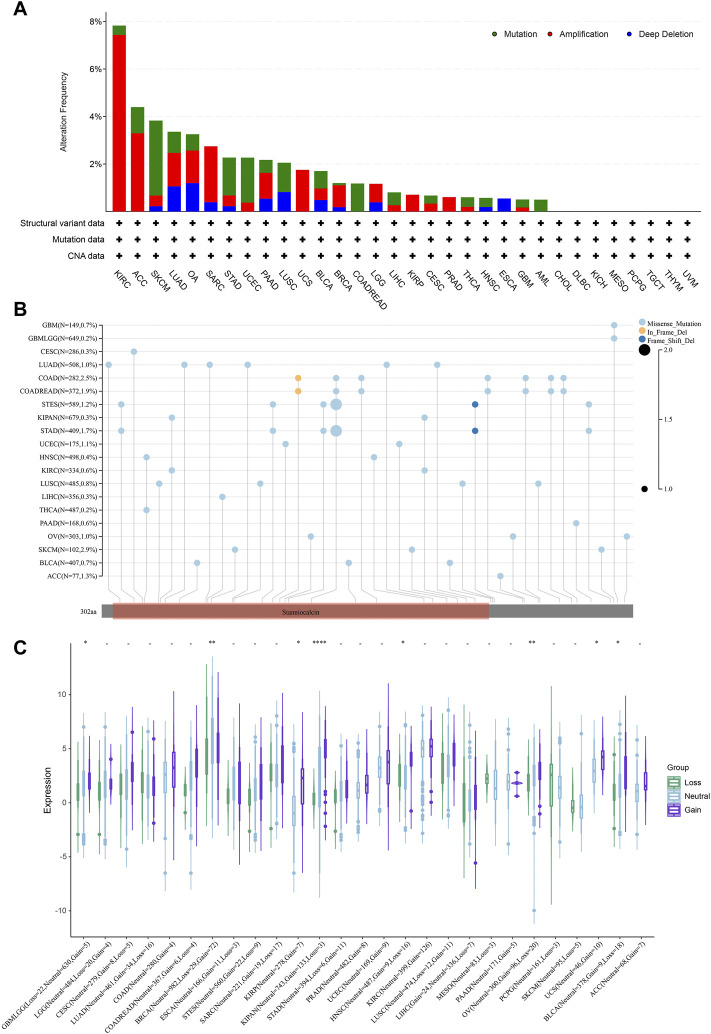
Mutation features of STC2. **(A)** Mutation features of STC2 in different tumor samples; **(B)** The types, sites and case percentage of STC2 genetic alteration; **(C)** The effects of copy number variation (CNV) on STC2 expression. *: *p*-value < 0.05; **: *p*-value < 0.01, ****: *p*-value < 0.0001.

It is known that the occurrence of tumors is greatly related to gene mutations, which cause the phenomenon of synonymous, missense, stop and frame shift mutations of codons, resulting in the gene expression alteration. We then analyzed the mutation features of STC2 in different tumor samples of the TCGA cohorts using cBioPortal database (Figure 9A). It showed that the highest alteration frequency of STC2 was presented in KIRC with amplification as the primary type. In UCS, KIRP, COADREAD, and LAML, only one type of alteration was found (UCS and KIRP: amplification. COADREAD and LAML: Mutation). The types, sites and case percentage of STC2 genetic alteration were further presented in Figure 9B. Three genetic alteration types including missense mutation, in frame deletion and frame shift deletion were observed, and it showed that missense mutation was the predominant type. In frame deletion was only observed in COAD and COADREAD, whereas frame shift deletion occurred in STES and STAD. In SKCM, 2.9% of cases were displayed missense mutation. Furthermore, the effects of copy number variation (CNV) on STC2 expression were investigated across various tumor types (Figure 9C). It revealed that STC2 expression was significantly correlated with CNV in tumors, such as GBMLGG, BRCA, KIRP, KIPAN, HNSC, OV, UCS, and BLCA.

### Correlation between the STC2 expression and tumor heterogeneity

**FIGURE 10:**
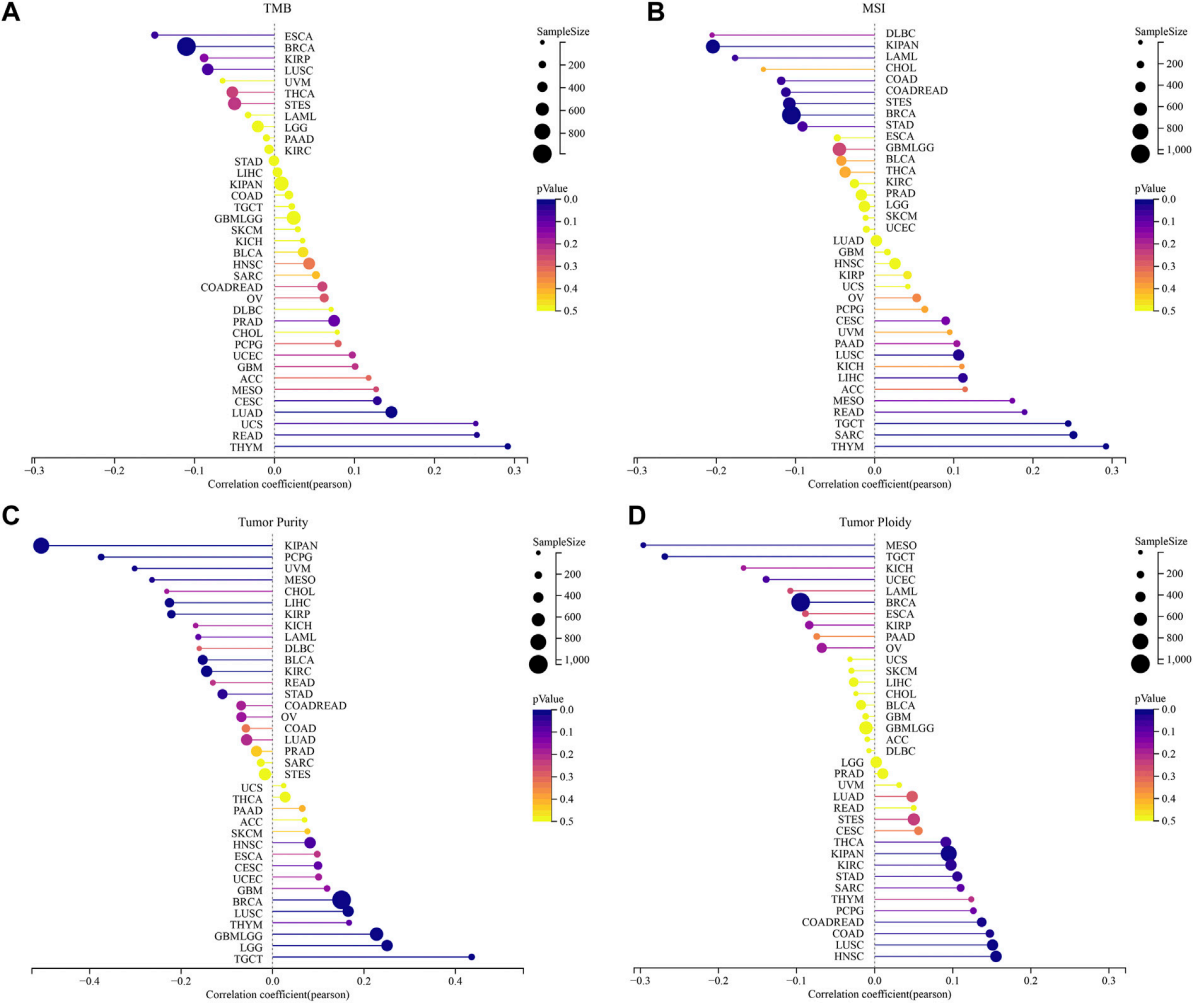
Correlation between the STC2 Expression and tumor heterogeneity. **(A)** Correlations between STC2 expression and TMB; **(B)** Correlations between STC2 expression and MSI; **(C)** Correlations between STC2 expression and tumor purity; **(D)** Correlations between STC2 expression and ploidy.

Analyzing the relationship between genomic heterogeneity and gene expression can provide more insight into tumors. Therefore, we investigated whether there were correlations between STC2 expression levels and TMB, MSI, tumor purity and ploidy (Figure 10). The results demonstrated that the expression of STC2 was related to TMB in six types of cancers, including CESC (R = 0.13, *p* = 0.03), LUAD (R = 0.15, *p* = 0.001), THYM (R = 0.29, *p* = 0.001), READ (R = 0.25, *p* = 0.02), BRCA (R = −0.11, *p* = 0.001), and ESCA (R = −0.15, *p* = 0.04) (Figure 10A). In 11 types of cancers, STC2 expression was associated with MSI, including SARC (R = 0.25,*p* < 0.001), LUSC (R = 0.11, *p* = 0.018), THYM (R = 0.29, *p* = 0.001), LIHC (R = 0.11, *p* = 0.03), TGCT (R = 0.24, *p* = 0.002), COAD (R = −0.12, *p* = 0.046), COADREAD (R = −0.11, *p* = 0.03), LAML (R = −0.18, *p* = 0.045), BRCA (R = −0.11, *p* < 0.001), STES (R = −0.11, *p* = 0.008), KIPAN (R = −0.20, *p* < 0.01) (Figure 10B). For tumor purity, STC2 expression positively correlated with five types of cancers and negatively correlated with nine types of cancers, like TGCT (R = 0.44, *p* < 0.01) and KIRP (R = −0.22, *p* < 0.001) (Figure 10C). Meanwhile, the expression of STC2 was associated with ploidy in 10 cancer types, with positive correlation in seven cancer types and negative correlation in three cancer tpyes, such as STAD (R = 0.11, *p* = 0.03), HNSC (R = 0.16, *p* < 0.001), MESO (R = −0.30, *p* = 0.001) and TGCT (R = −0.27, *p* = 0.001) (Figure 10D).

### Correlation between STC2 expression and tumor stemness

**FIGURE 11:**
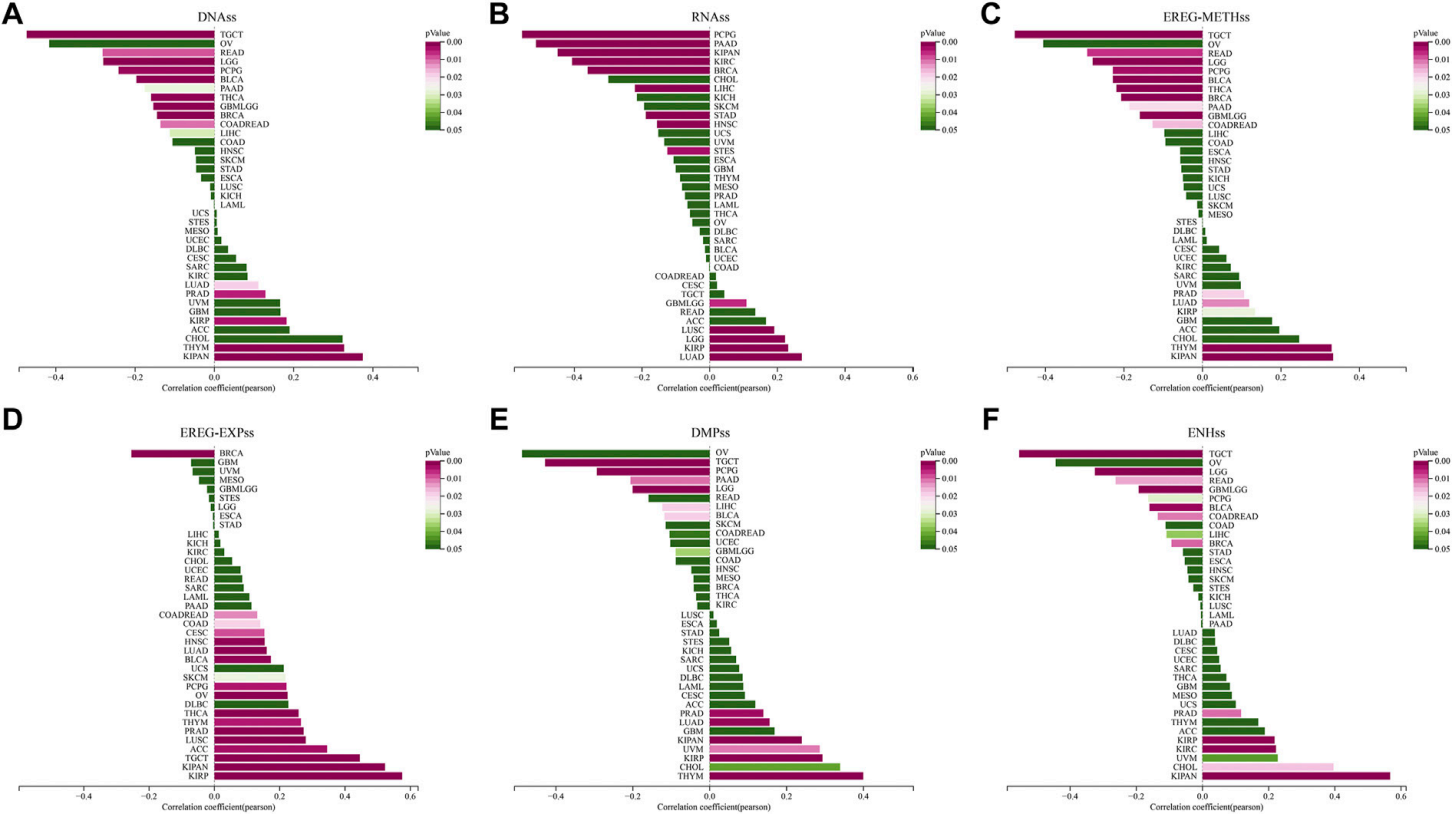
Correlation between STC2 expression and tumor stemness. **(A–F)** Relationship between STC2 expression and DNAss, RNAss, EREG-METHss, EREG-EXPss, DMPss and ENHss.

Cancer stem cells are involved in various processes of tumorigenesis. The stemness status significantly influences the direction and metastatic extent of cellular carcinogenesis. We estimated the relationship between STC2 expression and tumor stemness according to the previous study. The *p*-value and the correlation coefficient (R) of DNAss, RNAss, EREG-METHss, EREG-EXPss, DMPss and ENHss were showed (Figures 11A–F). Compared with those in other cancer types, the lowest correlation between STC2 expression and DNAss (R = −0.47, *p* < 0.001), EREG-METHss (R = −0.48, *p* < 0.001), DMPss (R = −0.43, *p* < 0.001) and ENHss (R = −0.55, *p* < 0.001) were found in TGCT. In EREG-EXPss estimation, only one genitive correlation was observed in BRCA (R = −0.25, *p* < 0.001). In KIPAN, STC2 expression displayed positive correlation with all of the stemness estimation expcept RNAss (DNAss: R = 0.37, *p* < 0.001. EREG-METHss: R = 0.33, *p* < 0.001. EREG-EXPss: R = 0.52, *p* < 0.001. DMPss: R = 0.24, *p* < 0.001. ENHss: R = 0.57, *p* < 0.001). In PCPG (R = −0.55, *p* < 0.001) and LUAD (R = 0.27, *p* < 0.001), the lowest and the highest correlation between STC2 expression and RNAss were estimated. Moreover, STC2 expression were most posetively associated with EREG-METHss (R = 0.33, *p* < 0.001) and DMPss (R = 0.40, <0.001) in THYM.

### Correlation between STC2 expression and RNA methylation modifications

**FIGURE 12:**
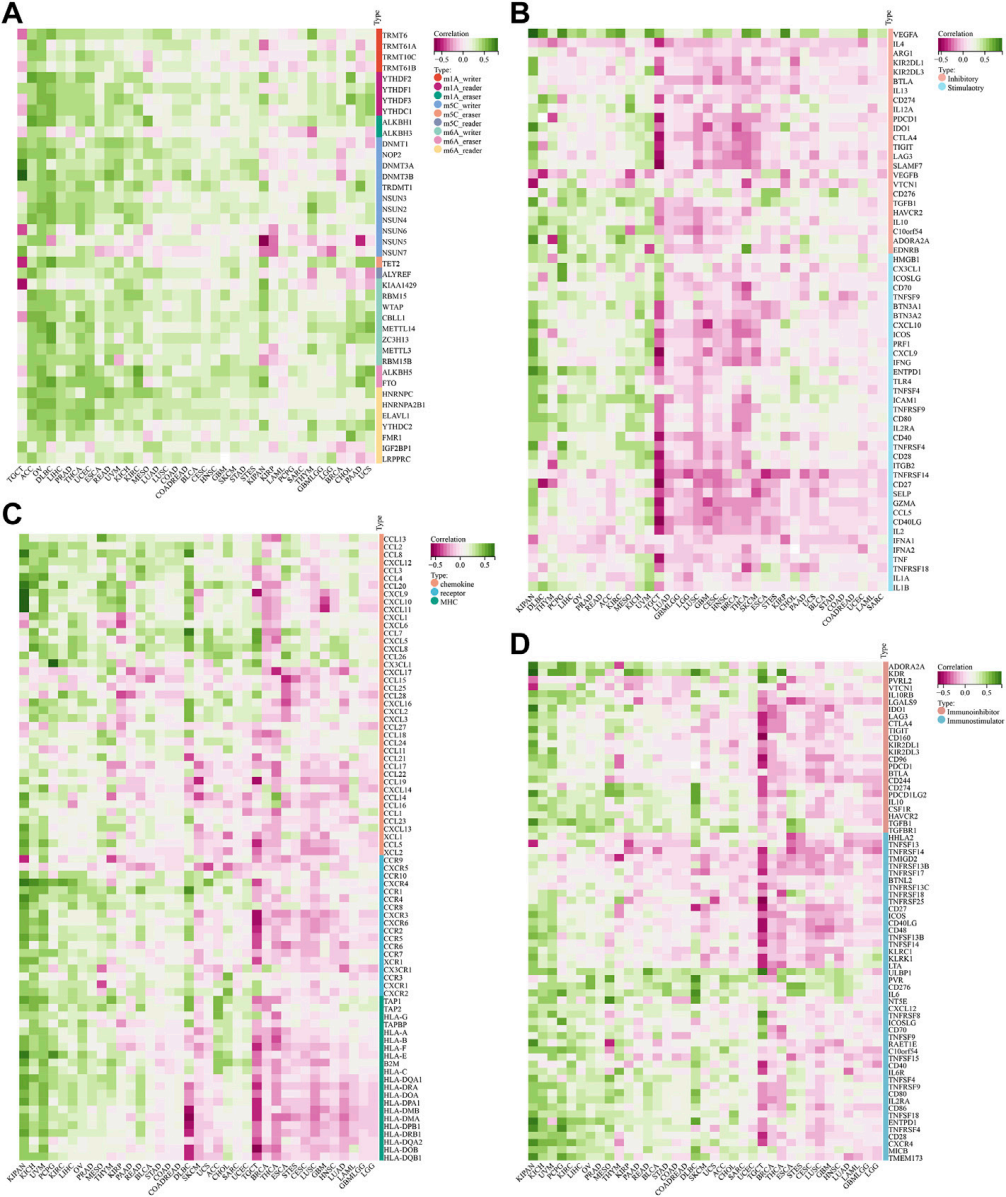
Correlation between STC2 expression and RNA methylation modifications. **(A)** Relationship between the expression of STC2 and three types of RNA methylation modifications; **(B)** Relationship between STC2 and immune checkpoints genes; **(C,D)** Immunological correlation of STC2.

In view of the important role of RNA methylation plays in the development of tumors, we assessed the relationship between the expression of STC2 and three types of RNA methylation modifications, including m1A, m5C, and m6A (Figure 12A). The results demonstrated that STC2 expression positively correlated with most RNA methylation genes across 37 tumor types. Especially in OV, it showed that STC2 expression was positively associated with all of the 44 genes related to three types of RNA methylation modifications.

Next, we estimated the association between STC2 with 60 genes related immune checkpoints (Figure 12B). The results demonstrated that STC2 exhibited a strong positively correlation with VEGFA across majority of tumors. Meanwhile, we also found that the expression levels of PD-1, PD-L1 and CTLA4 were positively associated with STC2 expression in most cancer types. Furthermore, we analyzed immunological correlation of STC2 (Figures 12C,D). The findings revealed that STC2 expression was associated with immunomodulators including chemokine, receptor, MHC, immunoinhibitor and immunostimulator. In KIPAN, UVM and LIHC, STC2 was positively correlated with a majority of the immunomodulators, whereas was negatively correlated with most of the immunomodulators in TGCT, BRCA and THCA.

## Discussion

Cancer has been the top killer of human all over the world. Among the numerous environmental factors that could contribute to cancer, PM2.5 is a particularly important risk factor. It has been reported that long-term exposure to air with PM2.5 concentrations higher than 10 μg/m^3^ leads to an increase in the prevalence of malignant tumors (Kim et al., 2015a). By using GSEA analysis, our study also further confirmed that PM2.5 exposure was correlated with a variety of lung cancers gene sets including non-small cell lung and small cell lung cancer. We subsequently identified a key gene, STC2, whose expression was up-regulated by PM2.5 and correlated with a poor prognosis in lung cancer. Importantly, our analysis found that STC2 expression was associated with multiple cancers. Therefore, we reasoned that PM2.5-induced STC2 may be a novel biological target for cancer immunotherapy.

STC is a class of secreted glycoproteins that consists of two family members, STC1 and STC2. STC1 is a paracrine protein, and its expression is higher in cancer tissues than in adjacent tissues, which is associated with tumor growth and cancer metastasis (Lin et al., 2021). STC2 protein is involved in the regulation of a variety of cell biological processes, and it similarly has a close relationship with tumorigenesis and development. STC2, as a secreted phosphor-protein, is detectable in serum and has good predictive value in several malignancies (Li et al., 2021). It has been reported that STC2 expression is elevated in tumor tissues and serum of patients with rectal cancer, and that elevated STC2 expression in tumor tissues and serum correlates with patients’ tumor pathological stage and poor survival (Huang et al., 2021). Our study also found that STC2 expression was up-regulated in COADREAD, COAD and READ, which may confirm STC2 serve as a potential tumor biomarker for the diagnosis and prognosis of rectal cancer. Researchers employed immunohistochemistry to analyze the clinical significance of STC2 and found that STC2 levels were higher in cancer tissues than in matched non-cancerous tissues (Pan et al., 2019). In our study, it was found that the expression of STC2 in most tumor tissues was significantly higher than that in adjacent normal tissues, except for KIRP, was lower in cancer versus adjacent normal tissues. Moreover, through the analysis of the overall survival period, we found that the high expression of STC2 is not only positively correlated with a poor prognosis for tumors like KIPAN and LUAD, but also play a protective role in LGG and GBMLGG. We also found that the expression of STC2 in Pan Cancer was also different due to different clinical features and pathological stages. In BRCA, BLCA, ESCA, COADREAD and READ, STC2 is expressed differentially between patients over 65 years of age and patients under 65 years of age, while this age correlation has not been observed in other cancers. In addition, we also found that there were significant gender differences in the expression of STC2 in eight types of tumors, including LAML, BRCA, KIRP, KIPAN, LUSC, LIHC, PCPG, and UVM. As for the relationship between the expression of STC2 and the clinical and pathological stages of tumors, we found that the differential expression of STC2 was related to stage, grade and TMN staging in majority of cancer types.

The tumor microenvironment is mainly composed of different cellular components including stromal cells, immune cells, and fibroblasts. Immune cells including granulocytes, lymphocytes and macrophages are involved in various immune responses such as tumor cell escape, immunosuppression and immune response (Sharonov et al., 2020; Ugel et al., 2021). Tumor associated immune cells may have tumor antagonizing or tumor promoting functions. We investigated the relationship between immune cell infiltration and STC2 gene expression in different cancer types, and found that STC2 expression was significantly correlated with the level of immune cell infiltration. In the analysis of B cells, T cells, neutrophils, macrophages, and dendritic cells, STC2 expression was found to be positively correlated with these immune cells in both PCPG and PRAD. While in TGCT and LUSC, the expression of STC2 was negatively correlated with these immune cells. Study has found that STC2 together with CD4^+^ T and CD8^+^ T cells infiltration influenced the prognosis of colon adenocarcinoma, and played a key role in the prediction of tumor patients at the molecular and cellular levels (Sun et al., 2020). Our study on colon adenocarcinoma revealed that STC2 expression was also associated with CD4^+^ T and CD8^+^ T cells. Also, study reported that the prognostic risk score was positively correlated with CD4^+^ T content, but not B lymphocyte, CD8^+^ T, neutrophil, macrophage, and dendritic cell content, in patients with esophageal squamous cell carcinoma (Liu et al., 2021). STC2 is a risk gene significantly associated with OS in patients with esophageal squamous cell carcinoma. The expression of STC2 was found to be negatively correlated with B lymphocytes in our study on ESCA, which is consistent with the above studies. But negatively correlated with CD4^+^ T cells and positively correlated with CD8^+^ T, neutrophils, macrophages, and dendritic cells. The reason for the discrepancy may lie in the different subtypes of ESCA. Researchers constructed a random forest classifier model based on the relative abundance of 24 immune cells against LUSC (Zheng et al., 2021). In our study of the relationship between the expression of STC2 and infiltration of immune cell subtypes, it was found that in LUSC, the expression of STC2 was negatively correlated with B cell subtypes but positively correlated with macrophage subtypes. It was demonstrated that lymphocytes and macrophages exerted different immune effects in LUSC.

Abnormal cell growth caused by gene mutations is important for tumorigenesis. Understanding the variety and impact of mutations in tumor associated genes in different tissues is essential for developing potential prevention strategies and proposing precise treatment regimens. Mutations in STC2 have a close relationship with the pathogenesis of head and neck cancer (Bajrai et al., 2021). Our study supported the above findings that the mutation types of STC2 in HNSC showed both missense and deletion mutations, and the percentage of missense mutations was relatively high. Some studies have found a close relationship between STC2 mutations and the onset and prognosis of liver cancer, especially in terms of overall survival and disease-free survival of patients (Kong et al., 2022). We found two types of mutations and amplifications of STC2 in liver cancer, with missense mutations as the predominant type, which confirmed the role of STC2 mutation in liver cancer. Tumor mutational burden (TMB), tumor microsatellite instability (MSI), tumor purity, and ploidy are strongly associated with tumor development, prediction of tumor prognosis, and choice of treatment for patients with cancer (Mann and Cheng, 2020; Cao et al., 2022). The investigators analyzed the effect of STC2 on the immune microenvironment of hepatocellular carcinoma using the tumor immune evaluation resource database and found that higher TMB was associated with a diverse immune microenvironment and poorer prognosis in hepatocellular carcinoma (Xie et al., 2020). STC2 expression was found to be associated with hepatocellular carcinoma tumor microsatellite instability in our study, which may provide a further therapeutic strategy for immunotherapy of hepatocellular carcinoma patients.

In recent years, with the rapid development of multiple genome sequencing technology, a large amount of stem cell data have been analyzed using advanced statistical techniques to explore the patterns that can be used to predict (Bai et al., 2020). Stem cell index was identified as a novel indicator of tumor development (Pan et al., 2019). Researchers found that metastatic tumors have a high stemness index and that the stemness index was negatively correlated with survival (Chowdhury et al., 2019). The cell stemness indicator is used as a prognostic indicator for a small number of tumors to help predict the risk of tumor recurrence and guide treatment (Li et al., 2017). Transfection of STC2 gene into stem cells was found to prolong cell survival time and protect cells from oxidative stress injury (Kim et al., 2015b). STC2 expressing stem cells exhibited higher cell viability and cell survival under sublethal oxidative conditions, illustrating that STC2 can be used to improve stem cell survival and long-term maintenance of stem cell therapy. Our analysis of the relationship between STC2 expression and cancer stemness identified a positive correlation between STC2 expression and DNAss、EREG-METHss and ENHss in KIPAN. It suggested that high expression of STC2 might be a poor prognostic factor for KIPAN.

RNA methylation and its related signaling pathways are involved in many biological processes, including cell differentiation, stress responses, and so on. Studies have found that RNA methylation has great potential as a new biomarker, or for targeted therapy of cancer (Law et al., 2008; Zhuang et al., 2020). Our findings also revealed that STC2 is involved in the methylation process in multiple tumors. We found by analysis that STC2 expression was positively correlated with all three types of RNA methylation modifications in OV, that STC2 expression was positively correlated with both m1A and m6A methylation modifications in COAD, and that STC2 expression was positively correlated with most m6A methylation modification related genes in PAAD.

## Conclusion

In conclusion, we explored the effect of PM2.5 on tumorgenesis, identified the regulated key gene STC2, and then systematically analyzed the pan-cancer information of multiple databases. However, there are limitations in this study. PM2.5 is a complex mixture of several components. It would be better to discover the specific component of PM2.5 for tumorgenesis and regulation of STC2. All results in this study are based on data mining. In the future, basic science and translational studies are further required to confirm these models. Taken together, the findings revealed that PM2.5-induced STC2 might be a potential prognostic and immunological biomarker for cancers related to air pollution.

## Data Availability

The original contributions presented in the study are included in the article/Supplementary Material, further inquiries can be directed to the corresponding authors.
