# Supplementary figures and images for: Transcriptome and pan-cancer system analysis identify PM2.5-induced stanniocalcin 2 as a potential prognostic and immunological biomarker for cancers
Source: Front Genet. 2023 Jan 6;13:1077615. doi: 10.3389/fgene.2022.1077615 (PMC9852732; doi:10.3389/fgene.2022.1077615)

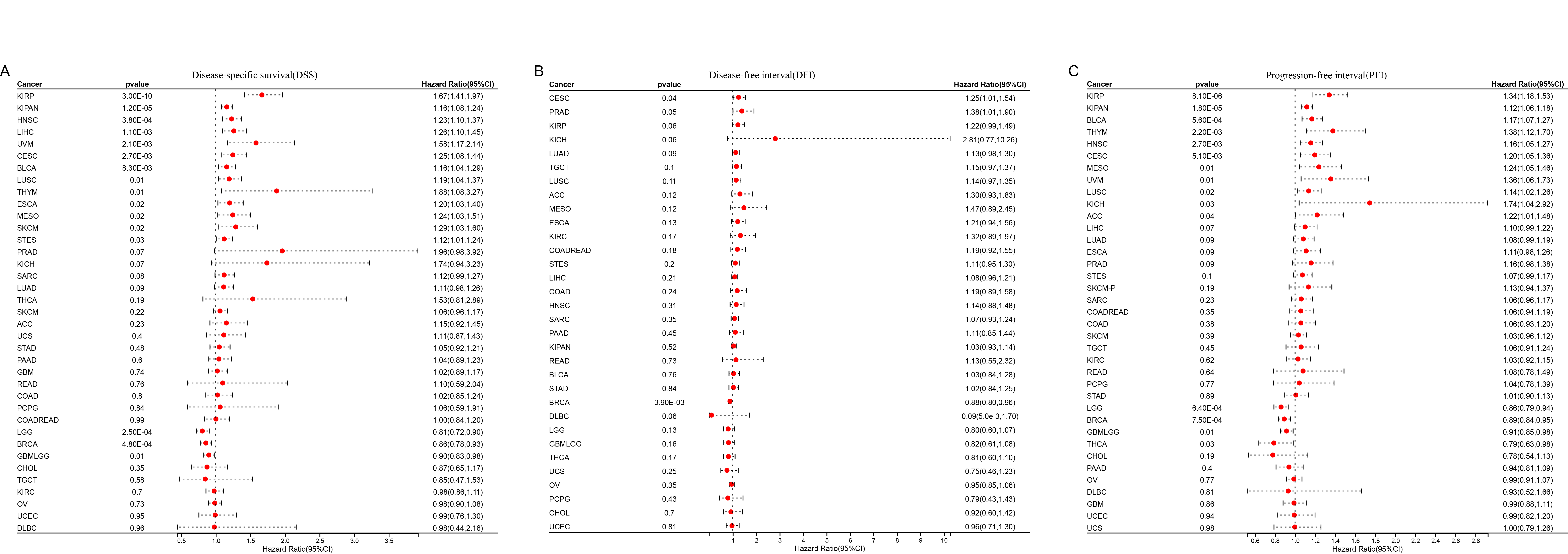

Supplement: Supplementary file 2 [file Image1.JPEG]
